# Synergistic actions of FGF2 and bone marrow transplantation mitigate radiation-induced intestinal injury

**DOI:** 10.1038/s41419-018-0421-4

**Published:** 2018-03-07

**Authors:** Byoung Hyuck Kim, Hee-Won Jung, Seok Hyun Seo, Hyemi Shin, Jeanny Kwon, Jae Myoung Suh

**Affiliations:** 1 Graduate School of Medical Science and Engineering, KAIST, Daejeon, Republic of Korea; 2 Division of Biological Warfare Preparedness and Response, Armed Forces Medical Research Institute, Daejeon, Republic of Korea; 3 Department of Radiation Oncology, Seoul National University College of Medicine, Seoul, Republic of Korea; 4 Biomedical Science and Engineering Interdisciplinary Program, KAIST, Daejeon, Republic of Korea; 5 Department of Radiation Oncology, Chungnam National University College of Medicine, Daejeon, Republic of Korea

## Abstract

Unwanted radiological or nuclear exposure remains a public health risk for which effective therapeutic countermeasures are lacking. Here, we evaluated the efficacy of fibroblast growth factor-2 (FGF2) in treating radiation-induced gastrointestinal syndrome (RIGS) incurred by lethal whole-body irradiation (WBI) when administered in conjunction with bone marrow transplantation (BMT). In vitro experiments indicated FGF2 treatment increased proliferation, reduced apoptosis, and upregulated AKT–GSK3β/β–catenin signaling in irradiated IEC-6 cells. We next established and analyzed mice cohorts consisting of sham irradiation (Group Sh); 12 Gy WBI (Group A); WBI with BMT (Group B); WBI with FGF2 treatment (Group F); and WBI with BMT and FGF2 treatment (Group BF). At 2 weeks post-irradiation, Group BF showed a dramatic increase in survival over all other groups. Intestinal epithelium of Group BF, but not Group B or F, showed augmented proliferation, decreased apoptosis, and preserved crypt numbers and morphology. Furthermore, Group BF maintained intestinal barrier function with minimal inflammatory disturbances in a manner comparable to Group Sh. In accordance, transcriptomic analyses showed significant upregulation of intestinal barrier and stem cell markers in Group BF relative to Groups A and B. Taken together, parenteral FGF2 synergizes with BMT to confer potent mitigation against RIGS.

The likelihood of exposure to high-dose radiation from nuclear plant accidents, terrorist attacks, or military conflicts continues to persist and increase. Leakage of radioactive material at the Fukushima nuclear power plant highlight the current risk of large-scale human exposure to potentially lethal doses of radiation^1,2^. Unfortunately, effective countermeasure agents to attenuate radiation injury in exposed individuals still remain a major unmet medical need. After acute irradiation, a complex array of clinical symptoms manifest depending on the dose, exposure rate, and type of radiation. First, hematopoietic syndrome can occur at doses of 1 Gy or more but adequate infection control, supportive care, and, more aggressively, bone marrow transplantation (BMT) can help subjects to regain hematopoietic function^3,4^. However, for subjects exposed to radiation doses high enough to induce acute RIGS, there are no effective treatments available currently, resulting in 100% mortality in about 2 weeks^5,6^. While BMT can readily support recovery from hematopoietic syndrome, its role in resolving radiation-induced gastrointestinal syndrome (RIGS) upon high-dose exposure remains controversial and of limited efficacy^7,8^. Radioprotectors, such as amifostine, are effective only when administered prior to radiation exposure^9^. Therefore, the development of radiation mitigators that can reduce toxicity after radiation exposure are essential to the arsenal of medical countermeasures for unexpected radiation incidents.

Intestinal stem cells located in crypts are required for maintaining tissue homeostasis and regeneration upon intestinal injury^10–12^. Previous studies demonstrate that crypt stem cell apoptosis is a primary cause for irreparable intestinal damage following radiation injury. Several growth factors, such as fibroblast growth factor-2 (FGF2), keratinocyte growth factor, and insulin-like growth factor, were shown to protect crypt stem cells from apoptosis and increase their survival after irradiation, albeit with limited efficacy, through mechanisms yet to be clarified^13–15^. Notably, the FGF family of secreted growth factors has been implicated in a broad range of biological processes including the maintenance and proliferation of stem cells, tissue remodeling, and homeostasis^16–20^. FGF2 has relatively higher thermostability in comparison to the closely related paralog FGF1 and exerts target cell effects through binding and activation of FGF receptor tyrosine kinases, typically in a paracrine or autocrine fashion. Endogenous FGF2 expression was shown to be upregulated in the small intestine after irradiation injury with suggested roles in preserving intestinal stem cells^21^. However, detailed physiological and molecular functions of endogenous FGF2 in intestinal tissue remodeling or regeneration after intestinal radiation damage remain unclear.

In clinical settings, the proposed dose range for a BMT referral is limited to 7–10 Gy as exposures exceeding 10 Gy negates any benefits of hematopoietic rescue by BMT due to fulminant RIGS^3,22^. Herein, we investigate whether FGF2 treatment alone or FGF2 treatment in conjunction with BMT, would counteract RIGS resulting from high-dose radiation exposure. Such approaches utilizing combined biologics may potentially extend the upper dose range for BMT referrals and lead to novel and effective treatment strategies for ameliorating RIGS.

## Results

### FGF2 improves cell viability and clonogenic potential after irradiation in vitro

**Fig. 1:**
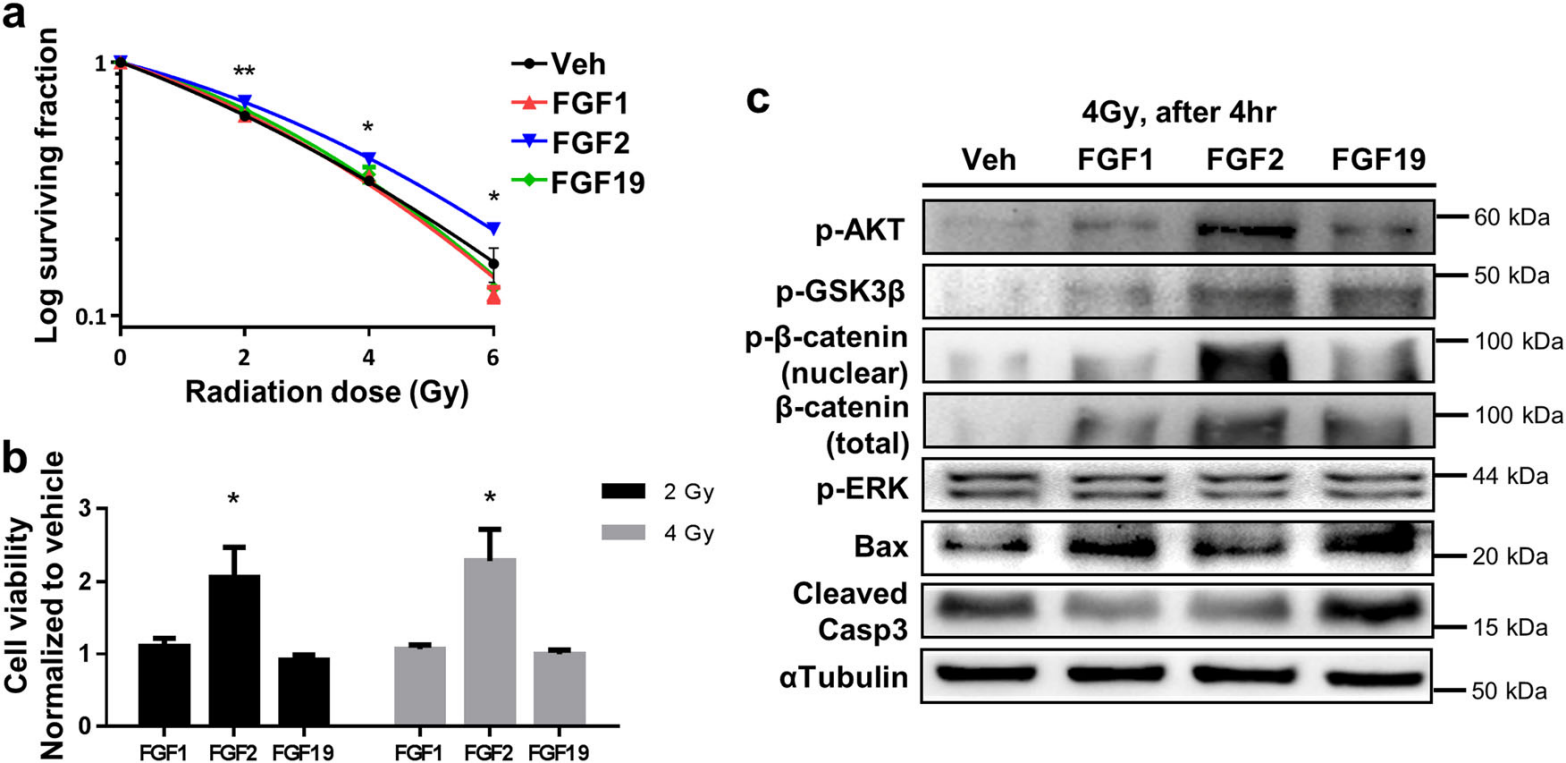
Evaluation of the radiomitigative effects of various FGF family proteins on irradiated IEC-6 cells. In all experiments, FGFs were added 30 min after irradiation. Asterisks indicate statistical significance between FGF2 and vehicle. **a** Clonogenic survival curve for irradiated IEC-6 cells. IEC-6 cells were treated with vehicle (PBS, Gibco), FGF1, FGF2, or FGF19 (each 10 ng/ml) for 24 h after the indicated doses of irradiation. *n* = 3 in each point. **b** Cell viability measured by CCK-8 assay 48 h after 2 Gy and 4 Gy irradiation, which were normalized to vehicle-treated group. *n* = 3 for each group. **c** Analyses of signaling pathways by Western blot with cell extracts from IEC-6 cells irradiated (4 Gy) and treated with FGFs for 4 h. Data are representative results from at least two independent experiments. Values are mean ± SEM; *p < 0.05, **p < 0.01

We tested multiple members of the FGF family for the potential to mitigate radiation damage in vitro using IEC-6 cells, which derive from rat small intestine. Among FGF family members tested, only FGF2 treatment for 24 h following irradiation significantly increased clonogenic cell survival as compared to other treatments (Fig. 1a). In addition to increased clonogenicity, cell viability also increased by FGF2 treatment as compared to other treatments (Fig. 1b). Next, we examined apoptotic signaling pathways and found that FGF2 treatment decreased the levels of Bax and cleaved Casp3. The decrease in apoptotic signals was accompanied by an increase in signaling through the AKT–GSK3β/β-catenin axis (Fig. 1c).

### FGF2 synergizes with BMT to enhance survival of mice exposed to lethal WBI

**Fig. 2:**
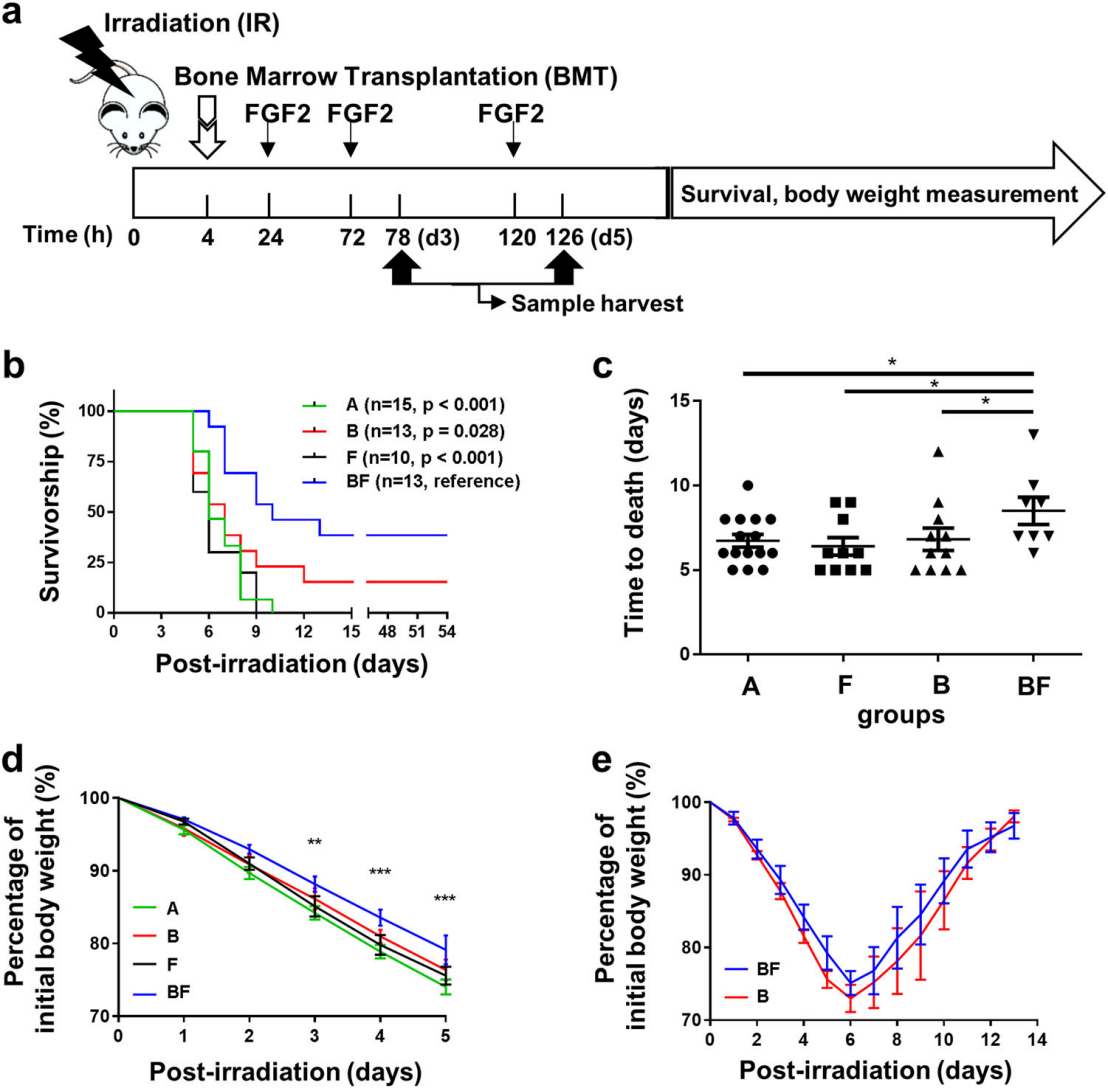
Effects of FGF2 injection with or without BMT on clinical parameters of C57BL/6 J mice after 12 Gy WBI. **a** Schematics of irradiation, FGF2 injection, BMT, and analyses at various time points. **b** Kaplan–Meier survival analyses of treatment groups; the number of mice per group and *p*-value compared with Group BF are shown in parentheses. **c** Survival time to death of mice that died within 2 weeks post-irradiation. **d** Body weight trends for mice that died within day 7 post-irradiation; *n* = 15 in each group. Asterisks indicate statistical significance between Group BF and Group A. **e** Body weight trends for mice in Group BF and Group B. Body weights of all surviving mice at the time of measurement are reflected in each data point; *n* = 13 per group on Day 0. Values are mean ± SEM. *p < 0.05, **p < 0.01, ***p < 0.001

To evaluate the in vivo effects of FGF2, either in the absence or presence BMT, a total of five groups of mice were established: sham irradiation (Group Sh); 12 Gy whole-body irradiation (WBI) without any treatment (Group A); WBI followed by syngeneic BMT (Group B); WBI followed by parenteral FGF2 treatment (Group F); and WBI followed by both syngeneic BMT and parenteral FGF2 treatment (Group BF) (Fig. 2a). Following a lethal dose of 12 Gy WBI, most mice from all groups died within 7 days; however, Group BF showed a significant increase in survival compared to all other experimental groups that received WBI (*p* < 0.001, Fig. 2b). Group BF also showed a significant increase in mean survival time of mice that died in the first 2 weeks after WBI (Fig. 2c). Body weight was measured daily for 13 days as a surrogate index for gastrointestinal function and general health of the mice. Irrespective of treatment group, body weight continuously decreased in all mice that died within the first 7 days after irradiation. Nevertheless, the trend of body weight loss was significantly dampened in Group BF relative to Group A (Fig. 2d). Among surviving mice, body weight decreased comparably in Group BF and Group B during the initial 5 days following irradiation, however, the rate of recovery to normal body weight from day 7 to 13 showed an increased trend for Group BF compared to Group B (Fig. 2e).

To assess the degree of hematological toxicity after irradiation, we performed complete blood count examinations. Analyses of subjects at day 5 post-irradiation demonstrated that severe leukopenia and mild anemia were present in all groups other than Group Sh (Supplementary Figure 1). Groups A and B did not significantly differ in terms of the white blood cell (WBC) count, hemoglobin level, and platelet count in peripheral blood (Supplementary Figure 1). These results indicate that transfused donor marrow had not undergone complete engraftment at the time of analysis. However, in Group BF, the administration of FGF2 did result in significantly higher hemoglobin levels (10.4 ± 0.3 vs. 11.9 ± 0.6, *p* = 0.008) and hematocrit values (36.0 ± 1.1 vs. 40.4 ± 2.8, *p* = 0.032) compared to Group B (Supplementary Figure 1). There were no significant differences in WBC and platelet count among all irradiated groups.

### FGF2 synergizes with BMT to restore intestinal epithelium damage from RIGS

**Fig. 3:**
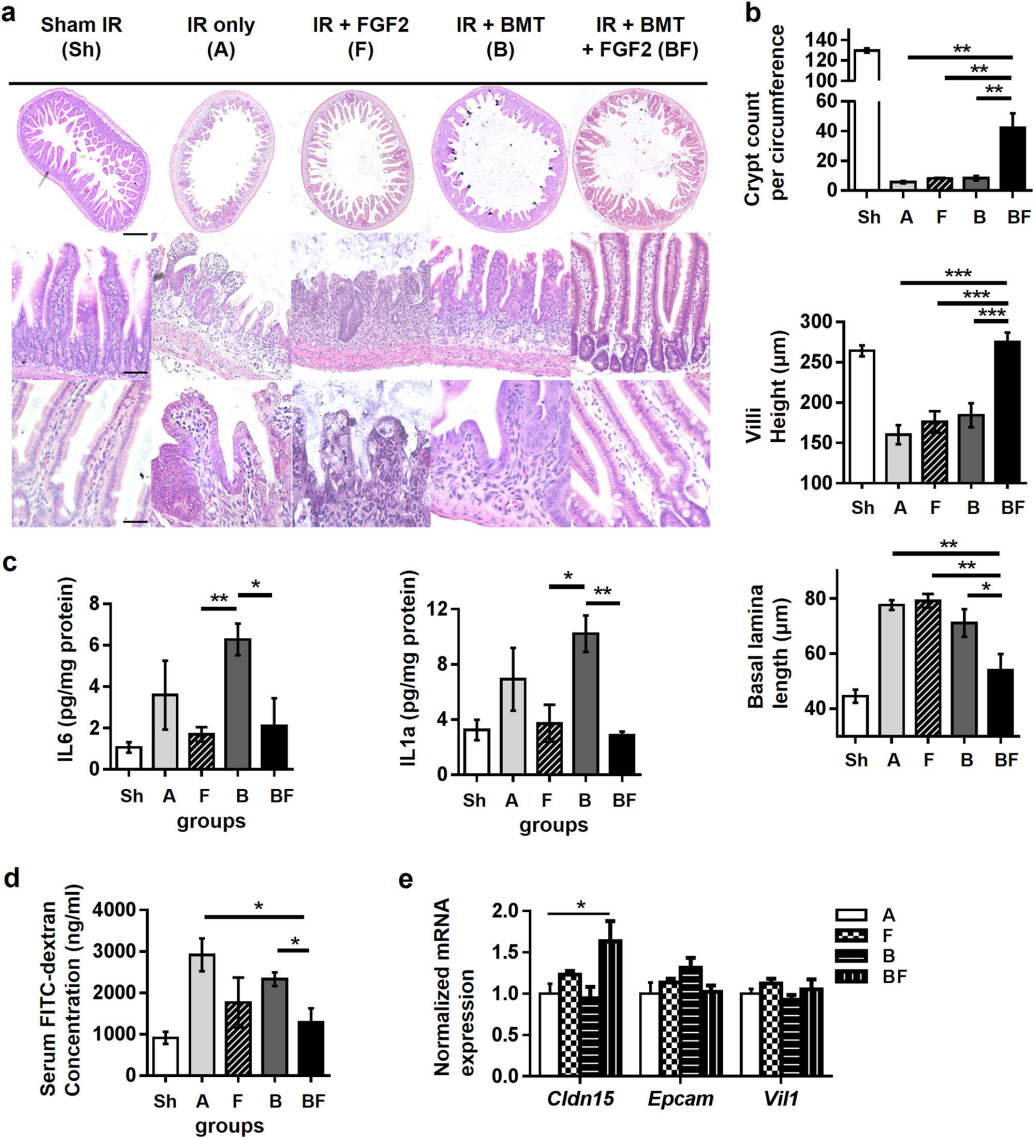
Effects of FGF2 injection with or without BMT on histologic parameters and intestinal inflammation. **a** Representative hematoxylin and eosin-stained sections of mouse small intestine at day 5 post-irradiation. Magnification ×40 (first row, scale bar 500 μm), ×200 (second row, scale bar 100 μm), and ×400 (third row, scale bar 50 μm). **b** Quantification of crypt counts per circumference, villi height, and basal lamina length. *n* = 5 in each group. **c** Intestinal IL-1α and IL-6 levels were measured in tissue lysates obtained at day 3 post-irradiation. Values are means ± SEM; *n* = 4 in each group. **d** In vivo intestinal permeability was determined by FITC–dextran serum concentration at 4 h after oral gavage; day 3 post-irradiation, *n* = 3 in each group. **e** *Cldn15*, *Epcam*, and *Vil1* expression levels at day 3 post-irradiation; *n* = 4 in each group. Values are mean ± SEM; *p < 0.05, **p < 0.01, ***p < 0.001

Since 12 Gy WBI resulted in the loss of crypts within 3 to 5 days after irradiation (Supplementary Figure 2), we examined histologic characteristics and functional permeability of intestines on days 3 and 5. In contrast to intestines from Group Sh, which showed normal tissue architecture, those from Group A, F, and B showed massive infiltration of inflammatory cells, ulceration with detachment of the mucosal layer, destruction of crypt/villi structure, and lengthened basal lamina (Fig. 3a). In striking contrast, Group BF showed sparing of intact crypt/villi structure accompanied by a greatly attenuated inflammatory response with levels comparable to that of Group Sh. Quantitative analyses of histologic parameters showed that Groups A, F, and B, in comparison to Group Sh, had decreased crypt counts per circumference (5.6, 8.2, 8.3 vs. 129.7), shorter villi heights (160.4, 176.0, 184.4 vs. 264.2 μm), and longer basal lamina (77.6, 79.2, 71.1 vs. 44.6 μm). In addition, Group BF was the least affected in terms of crypt counts while villi height and basal lamina length were comparable to those of Group Sh (Fig. 3b). Cytokine profiles revealed pro-inflammatory cytokines IL-1α and IL-6 from intestinal tissue lysates were reduced in FGF2-treated groups at day 3 post-irradiation (Fig. 3c). At 12 Gy WBI, intestinal permeability is known to be significantly increased due to compromised barrier function; however, compared to other treatments, the increase in intestinal permeability was remarkably attenuated in Group BF and nearly indistinguishable to Group Sh (Group BF vs. B, p = 0.038; Group BF vs. A, *p* = 0.027; Fig. 3d). These changes in Group BF were accompanied by enhanced expression of *Cldn15*, a major component of epithelial tight junction complexes (Fig. 3e). Interestingly, the mitigative effects against RIGS were not observed when FGF2 treatment was performed ex vivo to isolated donor bone marrow prior to transplantation (data not shown).

### FGF2 treatment leads to enhanced proliferation of crypt stem cells in damaged intestine

**Fig. 4:**
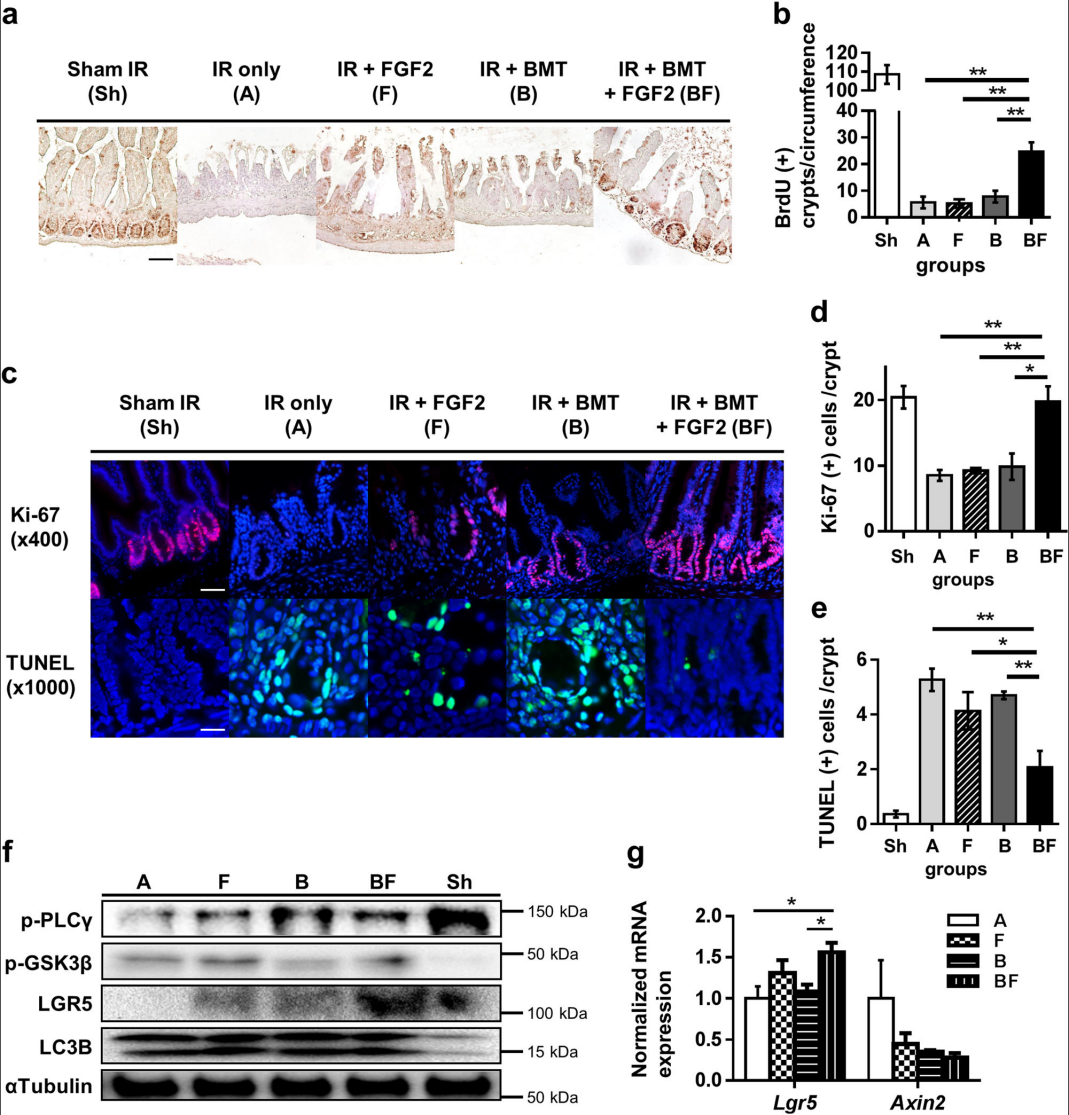
Effects of FGF2 treatment combined with BMT on intestinal crypt proliferation, apoptosis, and LGR5 expression. **a** BrdU incorporation measured by immunohistochemistry (magnification ×200, scale bar 100 μm) at day 5 post-irradiation. **b** Quantification of BrdU-positive crypts per circumference. At least five circumferences per mouse were counted; *n* = 3 in each group. **c** Ki-67 and TUNEL immunofluorescence staining (Ki-67: magnification ×400, scale bar 50 μm; TUNEL: magnification ×1000, scale bar 20 μm). **d**, **e** Quantification of Ki67- and TUNEL-positive cells per crypt at day 5 post-irradiation. At least 10 well-oriented crypts per mouse were counted; *n* = 3 in each group. **f** Intestinal tissue lysates were examined by Western blot at day 3 post-irradiation. α-tubulin was used as the loading control and LC3B was used as the positive control for radiation damage (autophagy). Data are representative results from at least two independent experiments. **g** *Lgr5* and *Axin2* expression levels at day 3 post-irradiation; *n* = 3 in each group. Values are mean ± SEM; *p < 0.05, **p < 0.01

We next examined the functional integrity of proliferating crypt structures in the small intestine, which is critical for normal homeostasis of intestinal tissue. The fraction of proliferating crypts, as determined by immunohistochemistry of bromodeoxyuridine (BrdU)-incorporated nuclei, increased significantly in Group BF compared to other groups (Fig. 4a, b). Therefore, the improved intestinal tissue integrity in Group BF (Fig. 3a) may be attributed to increased functionality of crypt stem cells that are required to replenish damaged intestinal epithelium following radiation injury. We further evaluated this notion by analyzing the pool of proliferating precursors with Ki-67 immunofluorescent staining. Similar to BrdU incorporation, Group BF intestines showed an increase in Ki-67-positive cells within crypt structures (Fig. 4c, d). Terminal deoxynucleotidyl transferase dUTP nick end labeling (TUNEL) staining also showed that crypt cell apoptosis in Group BF at day 3 was significantly reduced (Fig. 4c, e). Interestingly, protein levels of phospho-GSK3β (p-GSK3β), the inactive form of GSK3β which acts as negative regulator of the Wnt/β-catenin axis, and the intestinal stem cell marker *Lgr5* were both increased in Group BF compared to other groups (Fig. 4f). In agreement with these results, *Lgr5* mRNA expression was elevated in Group BF (Fig. 4g). Taken together, administration of FGF2 in conjunction with BMT enhances crypt stem cell proliferation after irradiation injury, possibly through inhibiting GSK3β activity and increasing *Lgr5* levels.

### FGF2 protects against radiation-induced alterations of inflammatory serum cytokines

**Fig. 5:**
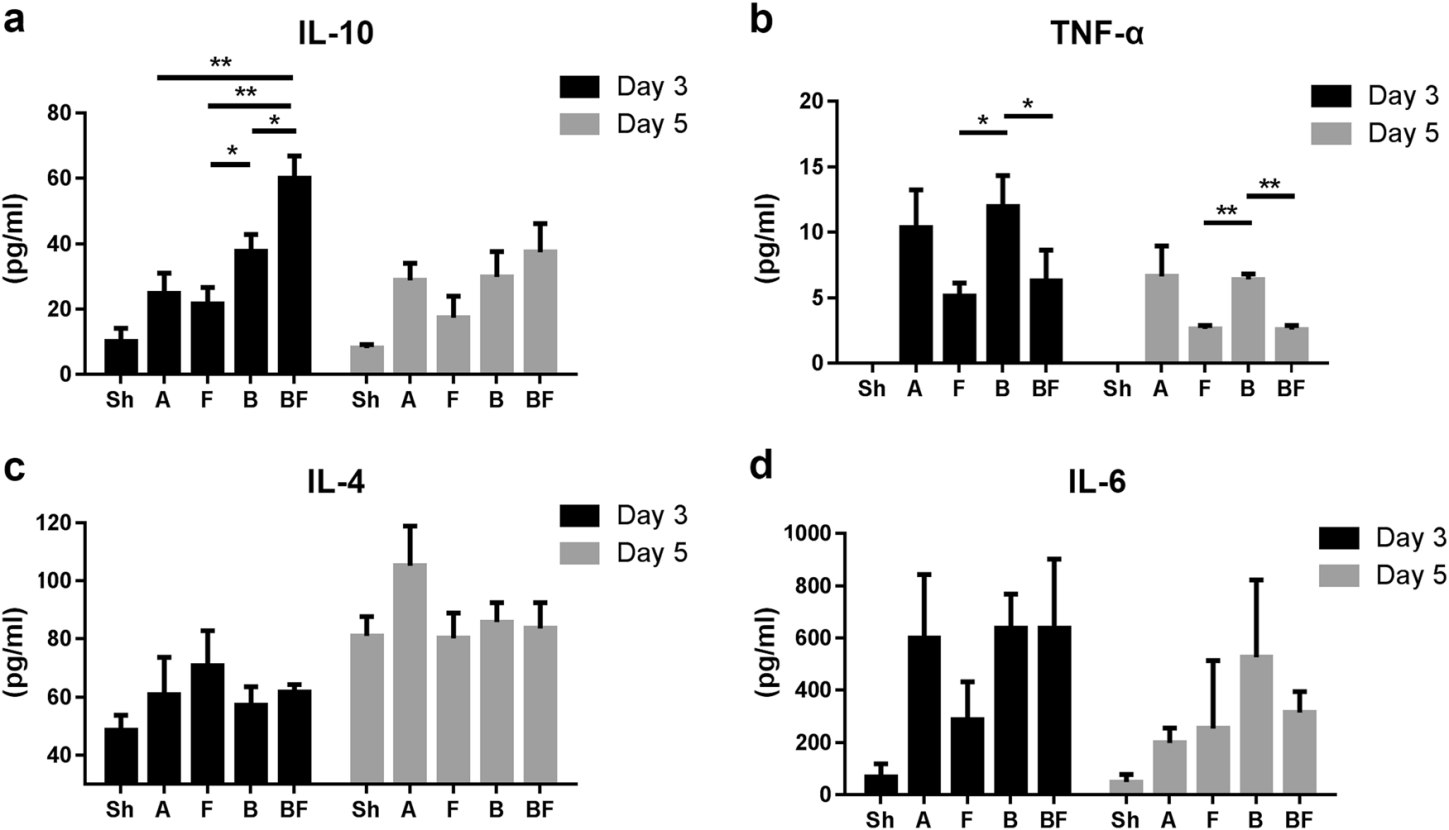
Effects of FGF2 treatment combined with BMT on radiation-induced alterations of serum cytokines. Serum cytokine profiles from mice on day 3 and day 5 post-irradiation. **a** IL-10. **b** TNFα. **c** IL-4. **d** IL-6. *n* = 3–6 in each group. Values are mean ± SEM; *p < 0.05, **p < 0.01

Irradiation induced a significant increase in serum cytokines IL-6, TNFα, and IL-10, consistent with expected radiation-induced systemic inflammation. Serum cytokine analyses on day 3 revealed an increase of anti-inflammatory cytokine IL-10 in Group BF, whereas pro-inflammatory TNFα decreased in FGF2-treated groups (Fig. 5a, b). IL-4 and IL-6 levels were not significantly different among treatment groups that received irradiation on both day 3 and day 5 (Fig. 5c, d). Other cytokines, such as IL-1α, IL-1β, IL-12p70, IL-2, and IFNγ, remained below the detection limit of the assay (data not shown).

### FGF2 treatment reveals transcriptomic signatures of reduced inflammation and restoration of intestinal tissue homeostasis

**Fig. 6:**
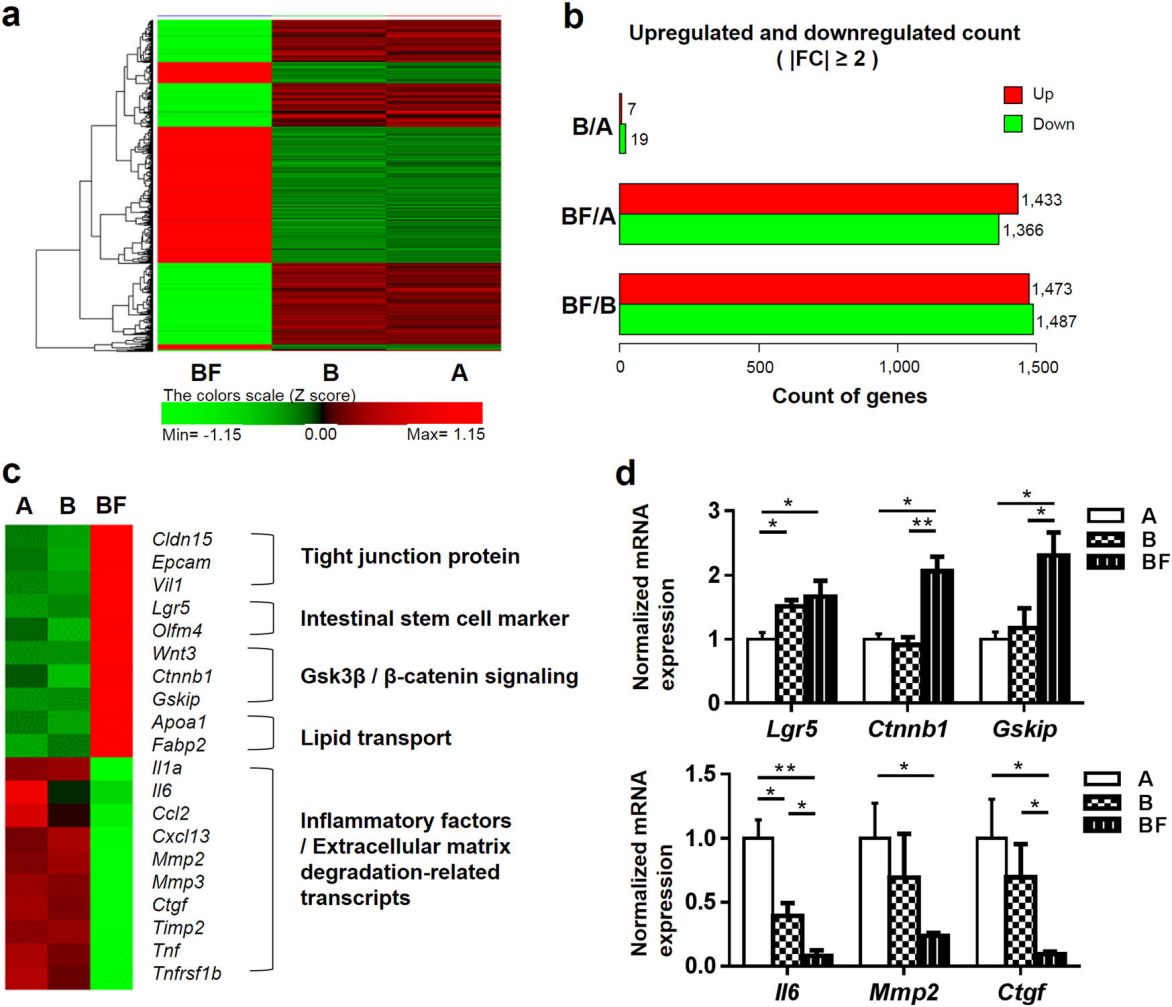
FGF2 treatment reveals transcriptomic signatures of reduced inflammation and restoration of intestinal tissue homeostasis in radiation-damaged intestines. **a** Heatmap of differentially expressed genes in intestinal tissue from indicated treatment groups (day 5 post-irradiation). Intestinal tissue RNA samples from three mice in each treatment group were pooled together for RNA sequencing. Red indicates higher normalized expression levels and green indicates lower expression levels. **b** Number of upregulated and downregulated genes (≥twofold change) between indicated treatment groups. **c** Heatmap of genes involved in intestinal tissue homeostasis and tissue inflammation. **d** qPCR validation of select genes from (**c**). *n* = 3 in each group. Values are mean ± SEM; *p < 0.05, **p < 0.01

To characterize molecular changes elicited by FGF2 treatment, we examined gene expression changes in intestinal tissues from three different treatment groups through mRNA-sequencing (RNAseq) analyses. Global gene expression analyses revealed that Groups A and B clustered together while Group BF showed a distinct pattern (Fig. 6a). We found 26, 2799, and 2960 genes with significant changes in expression levels (>twofold) in Groups A/B, A/BF, and B/BF, respectively (Fig. 6b).

Interestingly, in relationship to Groups A and B, Group BF showed higher expression levels of genes involved in intestinal tight junction protein (*Cldn15*, *Epcam*, and *Vil1*), intestinal stem cell maintenance (*Lgr5* and *Olfm4*), GSK3β/β-catenin signaling (*Wnt3*, *Gskip*, and *Ctnnb1*), and lipid transport (*Apoa1* and *Fabp2*) (Fig. 6c). Conversely, Group BF showed lower expression levels for genes encoding pro-inflammatory factors (*Il1α, Il6, Ccl2*, and *Cxcl13*) and extracellular matrix degradation-related transcripts (*Mmp2, Mmp3, Ctgf*, and *Timp2*). RT-qPCR analyses for representative genes showed similar expression changes obtained from RNAseq analyses (Fig. 6d). Therefore, transcriptomic analyses indicated a shift in global gene expression towards an enhanced recovery of damaged intestinal tissue by FGF2 treatment when provided in conjunction with BMT.

## Discussion

The underlying mechanisms of radiation-induced gastrointestinal injury are complex. Although BMT can rescue hematopoietic failure after acute high-dose radiation exposure, efficacious treatment strategies to ameliorate RIGS are yet to be established. A number of different growth factors have been implicated in the response to radiation injury; to this end, we sought to further investigate the biological role and therapeutic utility of FGF2 concerning RIGS. Survival analyses following high-dose irradiation revealed a role for FGF2 as a radiomitigator in mice receiving BMT. Analysis of lethally irradiated mice that received BMT followed by FGF2 treatment, Group BF, showed enhanced proliferation of intestinal crypt cells accompanied by histologic and functional recovery from intestinal radiation damage. On the other hand, intestinal epithelium of Group B displayed insignificant changes compared to Group A, indicating that BMT by itself cannot account for the improved intestinal tissue dynamics and functional recovery observed in Group BF. Therefore, FGF2 treatment ameliorates intestinal damage in RIGS through a newly identified synergism with BMT and thus suggests a novel treatment strategy to mitigate the effects of RIGS.

Numerous research efforts have been aimed towards the development of effective therapies to improve recovery from RIGS. Previous studies suggested that growth factors, including members of the FGF family, can have broad effects influencing the survival, proliferation, and differentiation of intestinal cells^13,21,23^. Endogenous FGF2 mRNA and protein in the small intestine increases 12 h after high-dose irradiation, reaching peak levels at 48–120 h after irradiation^21^. Radiation exposure induces expression of FGF2 in several cell lines in vitro as well^24,25^. These results suggest that endogenous FGF2 may play a role in the protective response to radiation exposure. Some studies show that exogenous FGF2 alone failed to increase crypt survival when administered after irradiation^21,26^, whereas others suggested that FGF2 as an independent treatment can improve crypt survival and restore gastrointestinal function, albeit with limited effect^9,27^. Others have reported that FGF2 injection a total of three times before and after radiation exposure (30 min before, 5 min before, and 30 min after) attenuated radiation-induced intestinal stem cell apoptosis through the Akt-p53 signaling pathway^28^. The aforementioned studies were performed under different experimental conditions, which may account for the varying outcomes. Together with our results, further investigations are warranted to determine the optimal course of FGF2 treatment for mitigating damage due to RIGS.

FGF2 has also been implicated in the attenuation of radiation-induced increase in intestinal permeability, which is considered an important cause of sepsis after WBI^29,30^. Results from our transcriptomic studies (Fig. 6c) indicate FGF2 may enhance barrier function through increased expression of *Cldn15*, a tight junction protein that also has a role in facilitating glucose absorption^31^. In addition, relative to Group A and B, Group BF showed decreased expression of many pro-inflammatory factors and MMPs in the intestine as well as a reduction in local IL-1α, IL-6, and serum TNFα cytokine levels. Therefore, FGF2 may exert effects against RIGS by enhancing intestinal barrier function and consequent resolution of both local and systemic inflammation.

Interestingly, FGF2 exerted radiomitigative properties in vivo, Group BF, as well as in vitro, FGF2-treated IEC-6 intestinal cells, as both showed decreased radiation-induced apoptosis. Analyses of intracellular signaling pathways suggested that the protective effects of FGF2 might work through the AKT–GSK3β axis, which in turn regulates nuclear β-catenin accumulation. GSK3β has been shown to induce apoptosis in various conditions, including DNA damage, while specific inhibitors of GSK3β are able to protect against radiation-induced damage^32,33^. On the other hand, GSK3β inhibition enhances β-catenin signaling by preventing its degradation, which in turn can activate a transcriptional program for the regeneration of the damaged intestinal epithelium^34,35^. Analyses of intestinal tissue from Group BF showed GSK3β inhibition and *Gskip*/*Ctnnb1* upregulation suggesting that the AKT–GSK3β/β-catenin signaling axis may be involved in the radiomitigative effects of FGF2. Further studies are required to delineate the molecular mechanisms underlying the synergistic actions of FGF2 and BMT in mitigating radiation injury.

A number of reports show that mesenchymal stem cells (MSCs) can facilitate the repair of tissue damage including intestinal injury and that MSCs have a potential for replacing injured intestinal stem cells^36–38^. In similar fashion, transplanted bone marrow-derived MSCs can promote engraftment of stem cells at the site of intestinal damage incurred by WBI, although the engraftment rates were quite low^39,40^. Other studies found that transplanted bone marrow cells could be detected in the recipient’s intestines as early as 2 days after transplantation^41,42^. In the present study, significant attenuation of radiation-induced intestinal damage occurred only when FGF2 was administered in combination with BMT. This suggests a cell type(s) and/or secreted factor(s) in the transfused bone marrow interacts to synergize with FGF2 to achieve the full range of effects observed in Group BF.

RIGS causes death at much earlier time points than death resulting from hematopoietic syndrome^43^. Our results suggest that FGF2 treatment, when combined with BMT, represents a viable intervention strategy for targeting the critical timeframe after radiation exposure but before RIGS-induced death occurs. Although BMT would help recover intestinal function once fully engrafted at several weeks post-irradiation, we speculate that FGF2 might enhance the incorporation of bone marrow-derived stem cells at an earlier time point during intestinal remodeling to offset the acute early stage mortality due to RIGS^8,44^. FGF2 has versatile functions in reducing local and systemic inflammation, supporting stem cell migration, engraftment, and proliferation in the intestine, all of which may contribute to FGF2-mediated mechanisms of radiomitigation^45,46^.

In summary, our current studies indicate that FGF2 combined with BMT can mitigate intestinal injury after acute high-dose irradiation. In treating radiation victims, the recommended therapeutic window for BMT is rather narrow (7–10 Gy) due to the limited efficacy of BMT at radiation doses exceeding 10 Gy. Our studies suggest that combining FGF2 treatment together with BMT could widen this window if translatable to humans. Further experiments are required to clarify the underlying mechanisms, optimal dose, treatment sequence, and potential adverse effects of combined therapy of FGF2 and BMT in RIGS. Nevertheless, given the paucity of available therapeutic strategies for RIGS, our findings point to a novel strategy to mitigate intestinal radiation injury and offer new insights into the pharmacologic actions of FGF2.

## Materials and methods

### Cell lines, recombinant proteins, animals, and irradiation

Rat intestinal epithelial cell line IEC-6 was obtained from the Korean Cell Line Bank (Seoul, South Korea) and maintained in DMEM with 10% FBS and 1% penicillin–streptomycin. Human recombinant proteins were purchased from Prospec (East Brunswick, NJ, USA). A total of 8–9-week-old male C57BL/6 J mice (Central Lab Animal Inc., Seoul, South Korea) were housed at room temperature under 12-h light–dark cycle and allowed access to water and chow ad libitum. All animal experiments were approved by the Institutional Animal Care and Use Committee at the Korea Advanced Institute of Science and Technology (KA2015-35). Mice and cells were irradiated at a dose rate of 2.16 Gy/min using a Cs-137 irradiator (Gammacell 3000 Elan, Best Theratronics, Ottawa, ON, Canada). Irradiation was performed without anesthesia on mice held in a 50-ml conical tube on a rotating turntable. Dosimetric quality assurance was performed using nanoDots (Al2O3:C) optically stimulated luminescence dosimeters (Landauer, Glenwood, IL, USA), which were read using a MicroStar OSL reader (Landauer, Glenwood, IL, USA).

### Clonogenic survival and CCK-8 viability assays

Clonogenic survival assays were performed as previously described^47^. Briefly, cells were plated immediately after irradiation at the same density into wells of a 6-well culture plate for each treatment group and cultured for 10–14 days to allow for colony formation. Colonies were fixed with methanol and stained with 0.5% crystal violet solution (Sigma-Aldrich, St. Louis, MO, USA). Colonies containing 50 or more cells were counted using ImageJ software (version 1.50, NIH, Bethesda, MD, USA), and the surviving fraction was calculated. For CCK-8 cell viability assays, cells were seeded in 96-well plates and treated with FGFs (10 ng/ml) for 48 h after irradiation. Viable cells were quantified by 450 nm absorbance measurement acquired 2 h after treatment with CCK-8 reagent (Dojindo Molecular Technologies, Gaithersburg, MD, USA)

### Mouse experimental groups and bone marrow transplantation

We performed BMT in mice 4–6 h after irradiation to evaluate the progression of gastrointestinal damage and subsequent recovery while minimizing the effects of hematopoietic system damage. Starting from 7 days before irradiation, the recipient mice were given acidified (pH 2.7) antibiotic water containing 1.1 mg/ml neomycin sulfate (N1876, Sigma-Aldrich, St. Louis, MO, USA) and 10^6^ U/l polymyxin B (P-1004, Sigma-Aldrich, St. Louis, MO, USA). The mice were kept on antibiotic water for 2 weeks after irradiation. The procedure of generating bone marrow-transplanted mice was based on previously published studies, with minor modifications^44,48^. Recipient mice received at least 5 × 10^6^ bone marrow cells via tail vein injection. For Group F and BF, mice were injected intraperitoneally with 1 mg/kg human recombinant FGF2 in three doses delivered 1, 3, and 5 days after irradiation while other groups were injected with vehicle (PBS, Gibco, Grand Island, NY, USA) at identical time points.

### Peripheral blood count

Whole blood was collected in EDTA-coated tubes (Greiner Bio-One, KremsmÜnster, Austria) from mice at the time of sacrifice. Peripheral blood cell counts were obtained using a veterinary hemocytometer XN-9000 (Sysmex Co., Kobe, Japan), according to the manufacturer’s instructions.

### Tissue harvest and histological analysis

Upon killing of mice, small intestinal tissues were quickly harvested from the interval spanning 5 cm distal to the gastroduodenal junction to 5 cm proximal to the ileocecal valve. Harvested intestinal tissues were trisected and the resulting segments were immediately processed as follows: fixation in neutral buffered formalin for histological analysis, immersion in RNAlater (Thermo Fisher Scientific, Wilmington, DE, USA) for RNA extraction, and snap freezing in liquid nitrogen for tissue lysate preparation. For histology samples, formalin-fixed tissues were embedded in paraffin and processed for routine histological examination. Tissue sections were deparaffinized, rehydrated, and used for immunohistochemistry and hematoxylin and eosin staining. For quantitative comparison, the proliferating crypts in the circumference of jejunal transverse cross-sections were counted. Proliferating crypts were defined as containing five or more adjacent chromophilic non-Paneth cells and a lumen. At least 10 circumferences were evaluated per mouse. To analyze morphological changes, villus height and basal lamina length of 10 enterocytes from the middle of randomly selected villi were measured. The lengths of the 10 longest villi in each slide were used for analysis in each sample.

### Immunohistochemistry and immunofluorescence staining

Proliferating cells in the jejunum were labeled with BrdU (ab142567, Abcam, London, UK), which was injected intraperitoneally into mice at 10 mg/kg 2 h before sampling. Briefly, deparaffinized and rehydrated sections were submerged in pH 6.0 sodium citrate buffer and heated to 95 °C for 15–30 min for antigen retrieval. Next, the sections were incubated with peroxidase-blocking solution (S2023, Dako, Carpinteria, CA, USA), followed by incubation with 1% normal goat serum for 30 min at room temperature to block nonspecific binding. Sections were washed with PBS three times and incubated with a primary antibody against BrdU (#5292, Cell Signaling Technology, Danvers, MA, USA) for 2 h at room temperature, followed by 1 h incubation with a species-specific Envision+ kit (Dako, Carpinteria, CA, USA), according to the manufacturer’s instructions. Liquid DAB + (K3467, Dako, Carpinteria, CA, USA) was used for visualizing. For immunofluorescence staining, anti-Ki-67 antibody (#12202, Cell Signaling Technology, Danvers, MA, USA) and DAPI (D9542, Sigma-Aldrich, St. Louis, MO, USA) were used. For TUNEL staining, In situ Cell Death Detection Kit (#1168-481-7910, Roche, Indianapolis, IN, USA) was used according to the manufacturer’s instructions.

### RNA extraction

Total RNA was obtained from tissues using TRIzol (Invitrogen, Carlsbad, CA, USA) according to the manufacturer’s protocol. RNA concentrations were measured with a NanoDrop spectrophotometer (Thermo Fisher Scientific). For RNA sequencing, RNA quality was assessed on an Agilent 2100 Bioanalyzer (Agilent Technologies, Palo Alto, CA, USA). RNA samples with RIN > 7.0, A260/280 > 1.5, A260/230 > 1.0, and rRNA ratio > 1.0 were used for subsequent analyses.

### Quantitative reverse transcriptase-PCR (qRT-PCR) analysis

After RNA quantification, 1 μg of total RNA was used to generate complementary DNA using HelixCript™ Easy cDNA Synthesis kit (Nanohelix, Daejeon, South Korea). To assess individual gene expression, real-time PCR was performed with Nanohelix premier qPCR premix (Nanohelix, Daejeon, South Korea) and ViiA 7 Real-Time PCR system (Applied Biosystems, Foster city, CA, USA). Relative quantification was based on the ΔΔCt method, and *RPLP0* gene was used as a reference control. PCR primers were designed using NCBI Primer-BLAST.

### RNA sequencing

RNA sequencing libraries were prepared using the Illumina TruSeq Sample Preparation Kit and sequenced on a HiSeq 2000 system (Illumina, San Diego, CA, USA), according to the manufacturer’s instructions. Sequencing was performed at a multiplexing level sufficient to generate >80 million reads per sample. The reads were aligned to the appropriate reference mouse genome (UCSC mm10) using TopHat. Aligned reads were converted to fragments per kilobase of transcript per million (FPKM) mapped reads values, calculated by the Cufflinks. After data preprocessing and QC processing, normalization was carried out based on the value of log (FPKM + 1) transformation^49^. When comparing two groups, we defined differentially expressed genes as those having a minimum of two-fold change in the expression of pooled samples. After analysis of RNA sequencing results, genes involved in intestinal homeostasis and tissue inflammation were chosen for further validation using qRT-PCR. Differential transcriptional profiles were presented as a form of heatmap using PermuteMatrix software^50^.

### Tissue and serum cytokine assays

Cytokines were analyzed using the Luminex Performance Assay kit (R&D systems, Minneapolis, MN, USA), according to manufacturer’s instructions. Briefly, duplicate serum samples or tissue lysates were added to a 96-well filter plate preconfigured with a panel of anti-cytokine antibodies covalently linked to unique polystyrene beads. Individual cytokines were identified and classified by their bead color using red laser excitation. Classified cytokines were quantified as a form of mean fluorescent intensity via green laser excitation. Standard curves for each cytokine were generated using the reference cytokine concentrations supplied by the manufacturer.

### Intestinal permeability assay

Serum concentration of FITC–dextran (FD4, Sigma-Aldrich, St. Louis, MO, USA) was used a measure of intestinal permeability as previously described^51^. Briefly, FITC–dextran was dissolved in PBS at a concentration of 100 mg/ml and was administered through oral gavage of 44 mg/100 g body weight. After 4 h, serum was collected by cardiac puncture. Fluorescence intensity of diluted sera was measured on a TriStar^2^ S LB 942 Modular Monochromator Multimode Reader (Berthold Technologies, Bad Wildbad, Germany). Serum from mice not administered with FITC–dextran was used to determine the background.

### Western blotting

Cells and tissues were lysed in modified RIPA buffer (50 mM Tris-HCl, 150 mM NaCl, 1% NP-40, and 0.5% sodium deoxycholate) and homogenized with FastPrep-24 (MP biomedicals, Orangeburg, NY, USA). Tissue homogenates were sonicated and insoluble debris was cleared by centrifugation. Protein concentrations of cleared lysates were quantified with Pierce BCA Protein Assay Kit (Thermo Fisher Scientific, Wilmington, DE, USA). Lysates were loaded on SDS-polyacrylamide gels and transferred onto polyvinylidene fluoride membranes. The membranes were blocked for 30 min at room temperature with 3% BSA in TBST and were then incubated with antibodies against p-AKT (Thr308, #9275, Cell Signaling Technology, Danvers, MA, USA), p-GSK3β (Ser9, #9331, Cell Signaling Technology, Danvers, MA, USA), p-β-catenin (PY489, Developmental Studies Hybridoma Bank, Iowa City, IA, USA), total β-catenin (ab32572, Abcam, London, UK), p-ERK (Thr202/Thr204, #9101, Cell Signaling Technology, Danvers, MA, USA), cleaved Casp3 (#9664, Cell Signaling Technology, Danvers, MA, USA), LGR5 (UMAB210, Origene, Rockville, MD, USA), α-Tubulin (sc-23948, Santa Cruz Biotechnology, Santa Cruz, CA, USA), Bax (sc-20067, Santa Cruz Biotechnology, Santa Cruz, CA, USA), and LC3B (#2775, Cell Signaling Technology, Danvers, MA, USA). Following incubation with HRP-conjugated primary host-specific secondary antibody (Cell Signaling Technology, Danvers, MA, USA), blots were visualized on a ChemiDoc™ XRS + System (BioRad, Hercules, CA, USA). α-Tubulin was used as the loading control.

### Statistical analysis

All values were expressed as the mean ± standard error of the mean (SEM), unless otherwise specified. The mean values between experimental groups were analyzed using Student’s *t*-test. Mouse survival curves were analyzed using Wilcoxon test. We considered *p* values < 0.05 as statistically significant. Data analysis was performed using GraphPad Prism version 6 (GraphPad Software, San Diego, CA, USA).

## Electronic supplementary material

Supplementary Figure 1

Supplementary Figure 2
