# Supplementary figures and images for: Synergistic actions of FGF2 and bone marrow transplantation mitigate radiation-induced intestinal injury
Source: Cell Death Dis. 2018 Mar 7;9(3):383. doi: 10.1038/s41419-018-0421-4 (PMC5841425; doi:10.1038/s41419-018-0421-4)

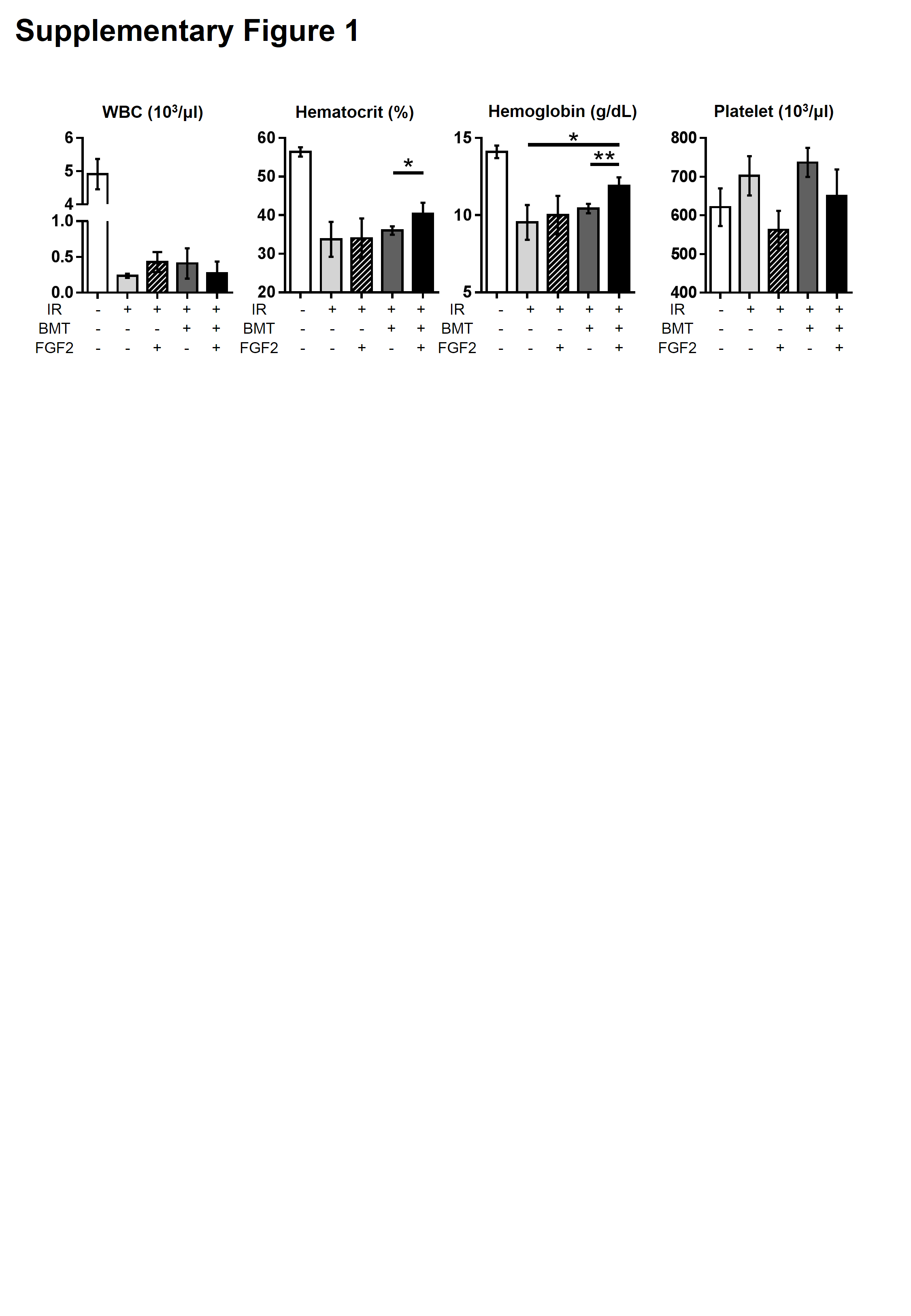

Supplement: Supplementary file 1 — Supplementary Figure 1 [file 41419_2018_421_MOESM1_ESM.tif]

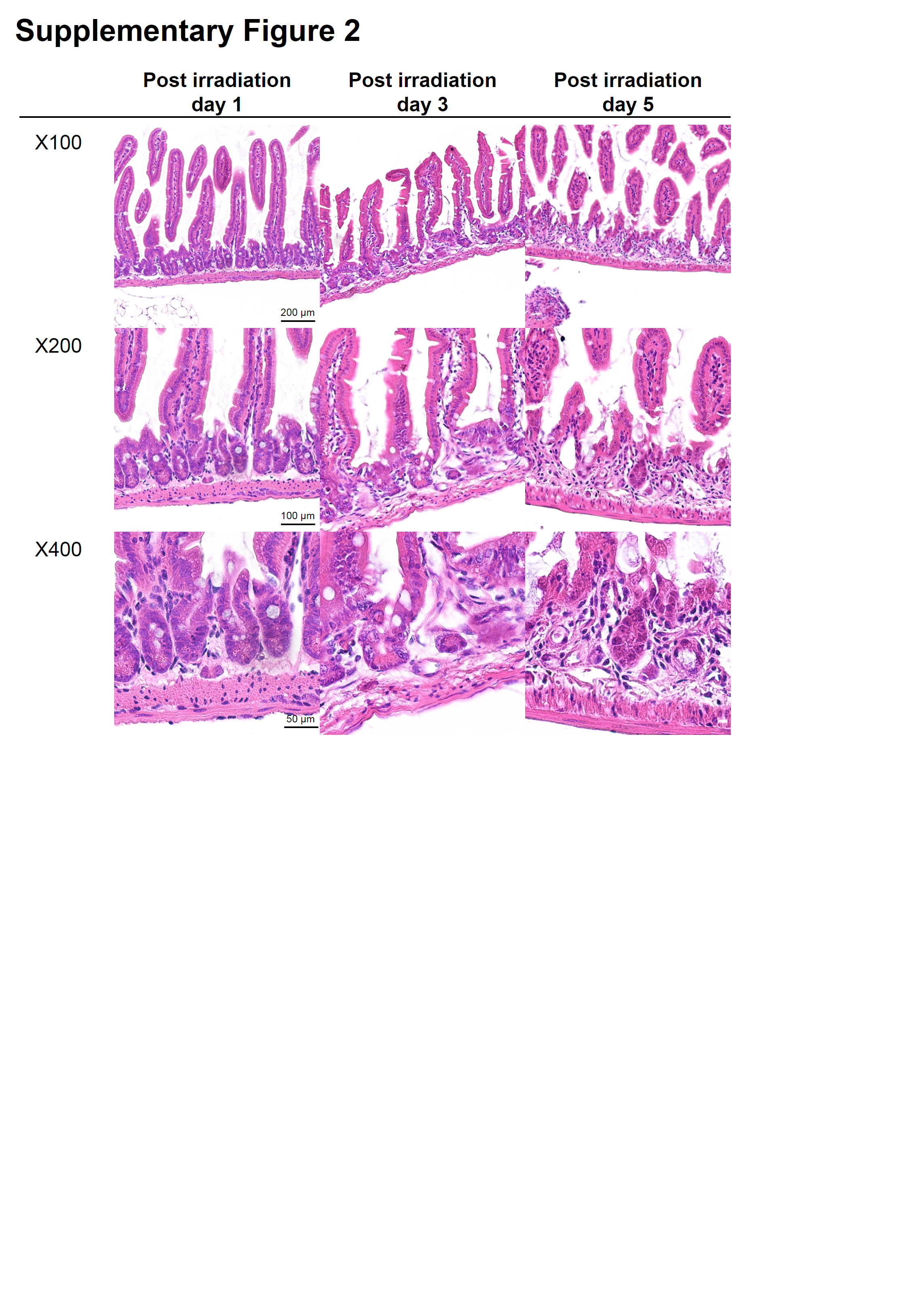

Supplement: Supplementary file 2 — Supplementary Figure 2 [file 41419_2018_421_MOESM2_ESM.tif]
